# Closure of live bird markets leads to the spread of H7N9 influenza in China

**DOI:** 10.1371/journal.pone.0208884

**Published:** 2018-12-12

**Authors:** Yin Li, Youming Wang, Chaojian Shen, Jianlong Huang, Jingli Kang, Baoxu Huang, Fusheng Guo, John Edwards

**Affiliations:** 1 Epidemiological Survey Division, China Animal Health and Epidemiology Center, Qingdao, Shandong, China; 2 Murdoch University Murdoch, Perth, Australia; 3 Hunan Provincial Center for Animal Diseases Control, Changsha, China; 4 FAO ECTAD China Office, Beijing, China; Nanjing Agricultural University, CHINA

## Abstract

Following the emergence of H7N9 influenza in March 2013, local animal and public health authorities in China have been closing live bird markets as a measure to try to control the H7N9 influenza epidemic. The role of live bird market (LBM) closure on the spread of N7N9 influenza following the closure of LBMs during March to May 2013 (the first wave) and October 2013 to March 2014 (the second wave) is described in this paper. Different provinces implemented closure actions at different times, and intensive media reports on H7N9 in different provinces started at different times. Local broiler prices dropped dramatically in places with outbreaks and more live chickens were transported to other LBMs in neighboring areas without human cases from infected areas when live bird markets were being closed. There were six clusters of human infection from March to May 2013 and October 2013 to March 2014 and there may have been intensive poultry transportation among cluster areas. These findings provide evidence that the closure of LBMs in early waves of H7N9 influenza had resulted in expansion of H7N9 infection to uninfected areas. This suggests that provincial authorities in inland provinces should be alert to the risks of sudden changes in movement patterns for live birds after LBM closure or increased publicity about LBM closure.

## Introduction

Since the first human case of influenza A (H7N9) in China in March 2013, there has been global attention on the development of this epidemic. There have been five epidemic waves of H7N9 influenza in humans in China since 2013 and a total of 1507 human cases (as of June 17, 2017) have been reported in mainland China [1, 2]. There were at least 732 cases recorded between October 2016 and June 2017. The sudden increase in human cases has caused global concern about the potential of a pandemic threat from H7N9 [3–5].

H7N9 infection in humans is associated with exposure to live poultry [6–8]. Poultry infections with the low pathogenic H7N9 influenza virus were confirmed soon after the first human case reports and virus isolates from human were highly homogenous with isolates from poultry [6]. Poultry infection was found mainly in live bird markets (LBMs) and H7N9 positive results were also detected on a few poultry farms. Surveillance results showed that yellow broiler [7] was the most infected type of bird in LBMs [8]. After genetic evolution over the last five years, highly pathogenic avian influenza (HPAI) H7N9 has recently been detected in the field [9–11].

National, provincial and local animal health and public health authorities have been taking active measures to try to control the emergency. One of the main measures taken by provincial and local government authorities has been the closure of LBMs in response to the detection of new human cases. This measure was considered successful because it appeared to decrease the number of human cases in these places for a short time [12]. A typical example was in Shanghai, where LBMs were closed in the middle of April 2013 and after two weeks there were no further human cases reported [13]. The National Health and Family Planning Commission (NHFPC) released an official guideline on 29 January 2014 and suggested that LBMs should be closed in an area where human cases were identified [14]. As a result, live poultry trade was suspended in many affected provinces and cities for weeks or even months and in some places longer term closures and restructuring of the live chicken markets were implemented. However, the traditional demand by Chinese people for consumption of freshly killed poultry seems difficult to change in the short term, and unauthorized live poultry trading has continued after the closure of local LBMs [15]. In the winter of 2016–17, there has been a sudden increase in reports of H7N9 human cases in rural areas and the virus appears to have been spreading more widely [5, 16]. The reasons for these changes are still not clear.

In this study, we describe the impact of the closure of LBMs, the resulting poultry movement patterns and the clustering of human infection in new areas in south eastern parts of China during the first two waves of H7N9 influenza. We hope this analysis can provide recommendations to reduce the spread of H7N9 influenza in China.

## Methods

### Data collection

Detailed information on all human cases from February 2013 to March 2014 was collected from provincial CDC websites, FluTrackers (http://flutrackers.com) and official media reports. This included onset date, confirmation date, report date and patient’s exposure location. Data on poultry positives were collected from the website of the Veterinary Bureau, Ministry of Agriculture. The poultry H7N9 surveillance data was generated from surveillance projects conducted by county, municipal and provincial veterinary authorities and was reported to the Veterinary Bureau, Ministry of Agriculture, and then was updated monthly. County level demographic data were from the *Tabulation of 2010 Population Census of The People’s Republic of China*. All the human case data were fully anonymized and no personal information would be publicly available.

Information on relevant market closures or policy announcements in Anhui, Jiangsu, Shanghai, Zhejiang, Fujian, Guangdong and Hunan provinces was collected from the relevant provincial Health and Family Planning Commission websites and from media reports. The price of broiler chickens in these provinces was obtained from a professional livestock industry website (http://www.xinm123.com/). In each province, prices of yellow broilers were collected from major wholesale LBMs and provincial averages calculated and updated on a daily basis. The number of relevant media reports in different weeks was using the major Chinese search engine “Baidu” with the key words: “H7N9” and “province name” in the word search (http://www.baidu.com/, search date: 5 June 2014). Outputs were re-checked to ensure that they were media reports relevant to the H7N9 topic. Duplicated reports on the same news from different media websites were included in the total count.

Poultry transportation data from a broiler trading platform in Anhui and 3 wholesale LBMs in Hunan province were collected by the China Animal Health and Epidemiology Center and the Hunan Provincial Center for Animal Disease Control (CADC). The broiler trading platform was owned by a commercial broiler company in Hefei city (capital city of Anhui province) and supplied more than 4.8 million live broilers to wholesale LBMs in other provinces in 2013. The LBMs were in Shaoyang City, Loudi City and Chenzhou City, the locations of 62% (13/21) of human cases in Hunan province in the first two waves. The data from the company covers the period from February to May in 2013; the data from the 3 LBMs covers the period from 1 January to 10 February 2014.

### Data analysis

LBM closures, relevant actions, changes in relevant media reports, prices and volume of transported live poultry to cities in Hunan province were described in a timeline using Microsoft Excel 2007 version (Microsoft Corporation, Redmond, WA). The poultry transportation data from the three wholesale LBMs in Hunan province, difference in daily importation volumes in different periods were analyzed with t test. Proportions of farmer patients among all the patients in the first epidemic wave (February to May 2013) and the second epidemic wave (October 2013 to March 2014) were compared with chi-square test. All statistical analyses were done with PASW Statistics 19.0 (SPSS Inc., IBM Corporation, Somers, NY).

Maps of human cases, poultry infection (virological positives), LBM contamination, transport direction for poultry and human case clustering, were created using ArcGIS 9.3 (ESRI Inc., Redlands, CA, USA). The imported human cases were marked on the map according to their exposure locations.

Clustering analysis for human cases was done with SaTScan v9.3 (Martin Kulldorff, Boston, USA), using data of human cases from February 2013 to 31 March 2014 and county level demographical data. Retrospective Space-Time analysis was conducted, using the Discrete Poisson model. For the spatial window, the maximum spatial cluster size was 10 percent of population at risk and 50 km in distance; for the temporal window, the minimum temporal cluster size was three days and the maximum 15 days.

## Results

### LBM closure in the second wave of H7N9 influenza

**Fig 1:**
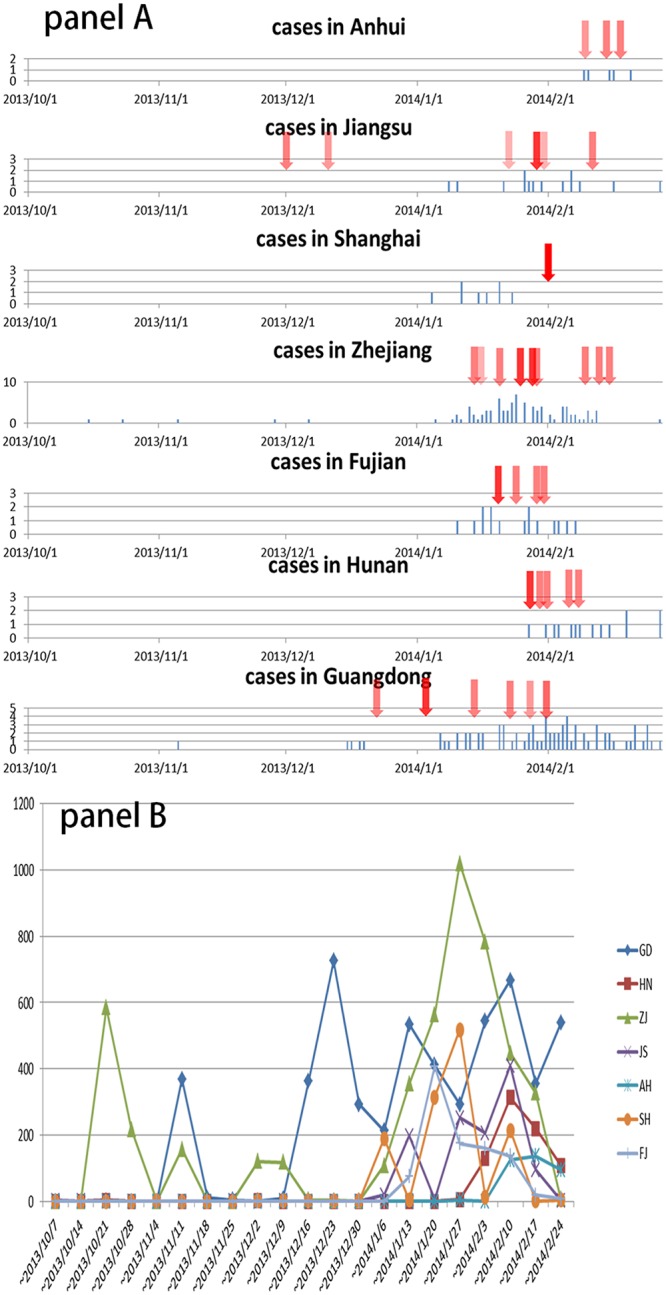
The epi-curves of human cases and timing of official LBM closure actions in different provinces (A) and the number of relevant media reports about H7N9 (B) in different provinces from 15 October 2013 to 24 February 2014. Abbreviation reference: GD: Guangdong; HN: Hunan; ZJ: Zhejiang; JS: Jiangsu; AH: Anhui; SH: Shanghai; FJ: Fujian.

Different provinces had human cases at different times, and LBM closure or relevant policy releases occurred at different times (Fig 1A and S1 Table). According to the variety of measures on LBMs closure and different geospatial coverages of each policy implementation, the magnitude of the impact induced by each action was determined by the authors (S1 Table). In Fig 1, each action was colored differently, according to the magnitude of impact: the bigger the impact, the darker the color. For example, Jiangsu province commenced a LBM closure policy from December 2013, while the neighboring Anhui province and Shanghai city took actions after February 2014. In Hunan province, actions were taken much later because the first human infection did not occur until late January 2014.

### Media reports and broiler price changes

Media reports about H7N9 influenza peaked in different provinces at different times. The included media reports were released much earlier in Guangdong and Zhejiang provinces than in the other provinces (Fig 1B). Following these peaks of media reporting, the relative broiler prices between neighboring provinces were disturbed. The average price of yellow broilers in Guangdong almost was always higher than the price in Hunan during 1 November to 9 December 2013. However, the price differential between these two provinces changed after late December 2013, when a peak of relevant media reports started in Guangdong on 9 December 2013 and Guangdong authorities started to close local LBMs on 23 December 2013 (Fig 1 and S1 Fig).

### Changes in live poultry movements in the first two waves

**Fig 2:**
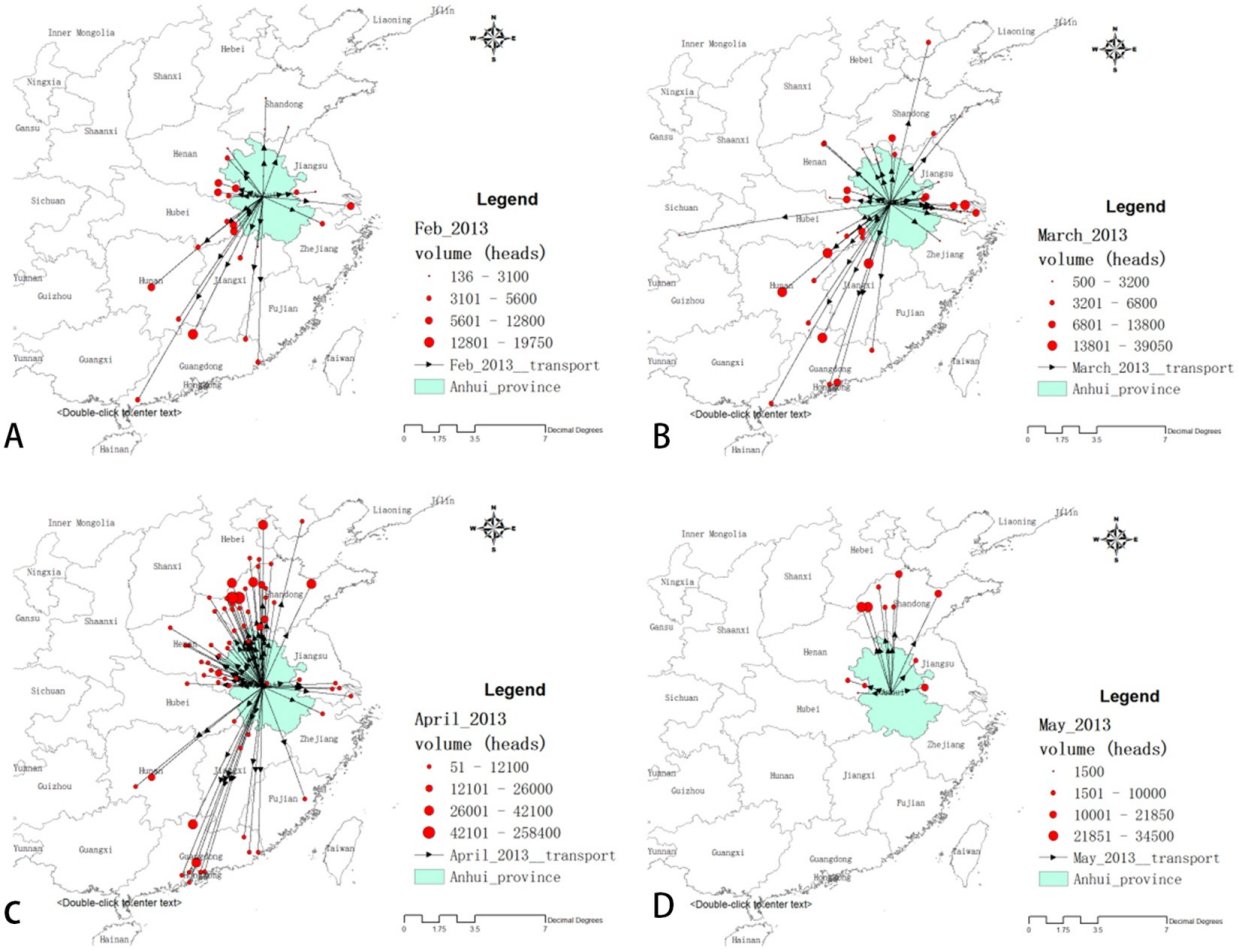
Transport from the broiler trading platform in Anhui province before and after the emergence of H7N9 influenza in 2013. (Monthly transport directions from the broiler trading platform in Anhui province during February to May 2013 were shown in A-D: A: February; B: March; C: April; D: May).

Poultry movement from the broiler-trading platform in Anhui was disturbed when H7N9 influenza emerged in March 2013. Before then, these broilers mainly went to Shanghai, Hubei, Hunan and Guangdong provinces. In April 2013, the volume of yellow broilers sent to eastern parts of China (especially big cities like Shanghai and Hangzhou city) decreased and movements to more northerly provinces (mainly to Shandong, Hebei and Henan provinces) increased (S2 Table). The number of transport destinations also increased from 44 destination counties in March to 69 destination counties in April. In May 2013, most of the yellow broilers were sent to Shandong province and almost no yellow broilers were sold to south and eastern China and trade volume dropped sharply (Fig 2).

**Fig 3:**
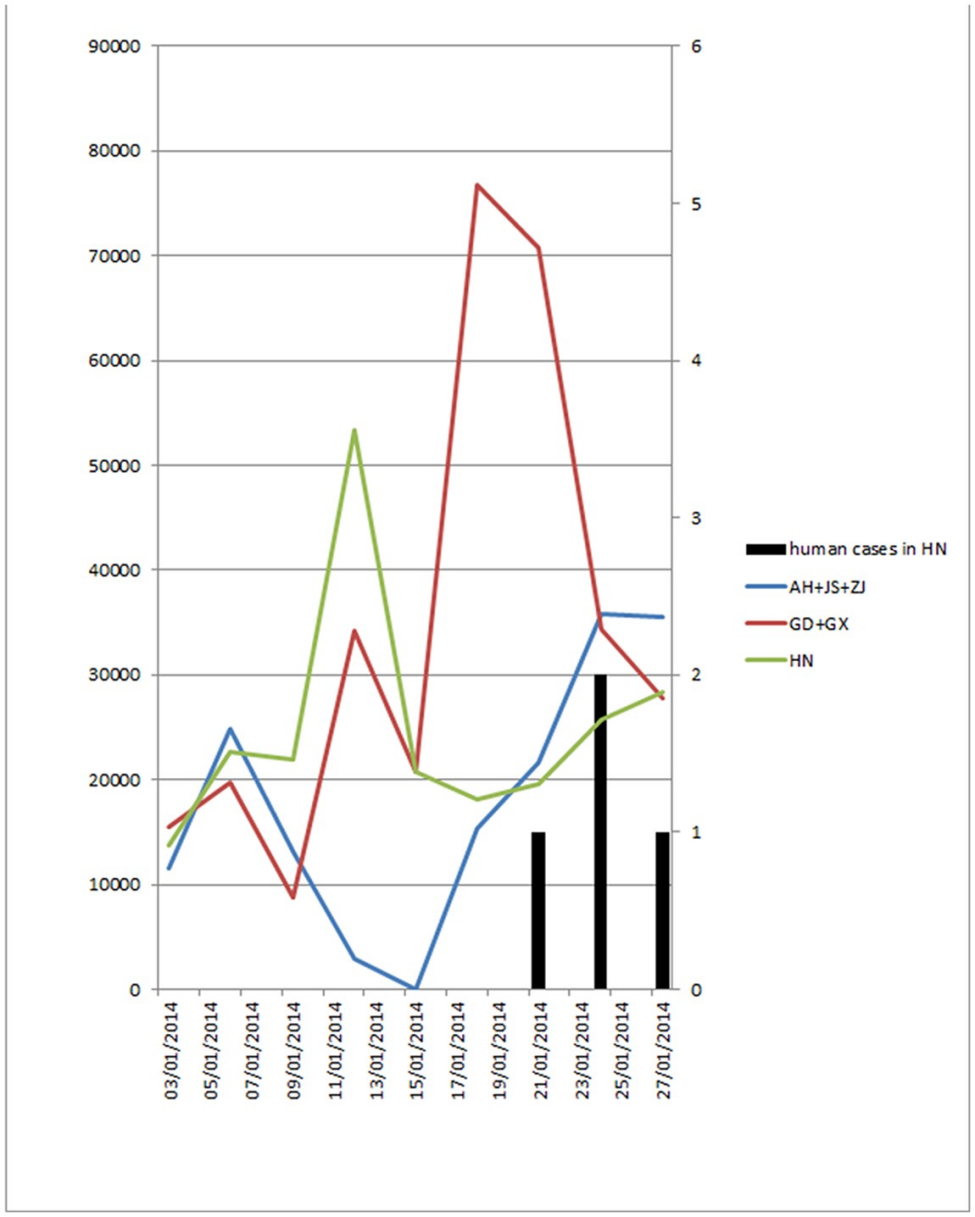
Changes in the volume of imported poultry from infected provinces and volume from local sources in 3 wholesale LBMs in Hunan province in January 2014.

According to data on live poultry importation (mainly chickens) for the three wholesale LBMs in Shaoyang, Chenzhou and Loudi cities in Hunan province, poultry were imported from infected provinces, including Guangdong, Guangxi, Anhui, Jiangsu and Zhejiang province between 1st January to 10th February 2014, but no poultry from Fujian province were imported to the three LBMs. We considered Guangdong and Guangxi as one group, and Anhui, Jiangsu and Zhejiang as the other group, because these two areas were the main infected areas in the first two waves. It shows that there were increases in poultry importation from these two infected areas before the onset date of the first human case in Hunan Province in winter of 2013 (Fig 3).

The greatest increase in poultry imports from South China (Guangdong and Guangxi provinces) was from 16 to 23 January 2014 and this immediately preceded the first occurrence of human cases of H7N9 influenza in Hunan Province. The average daily importation volume for the period was 21,602 birds, while the average daily importation volume at other times was only 5,716. The difference in daily importation volumes for the two periods is statistically significant (t test, p<0.01). The main increase in poultry imports from East China (Anhui, Jiangsu and Zhejiang province) was from 20 to 26 January, and the average daily importation volume for the period was 12,604 birds, while the average daily importation volume for the other days in January 2013 was only 2,934 birds. The difference in daily importation volumes of the two periods is also statistically significant (t test, p<0.01).

### Spread and clusters of H7N9 human cases in the first two epidemic waves

According to the timeline of LBM closure actions, each wave can be divided into an early stage and a late stage. The rough cut points for the 2 waves were 15 April 2013 and 23 January 2014 separately. Human cases spread to neighboring provinces late in the first two waves (S2 Fig). In the first wave, farmers occupied 21% of the total human cases and in the second wave, the percentage of farmer cases rose to 31%. This increase is statistically significant (Chi-square test, P = 0.03).

**Fig 4:**
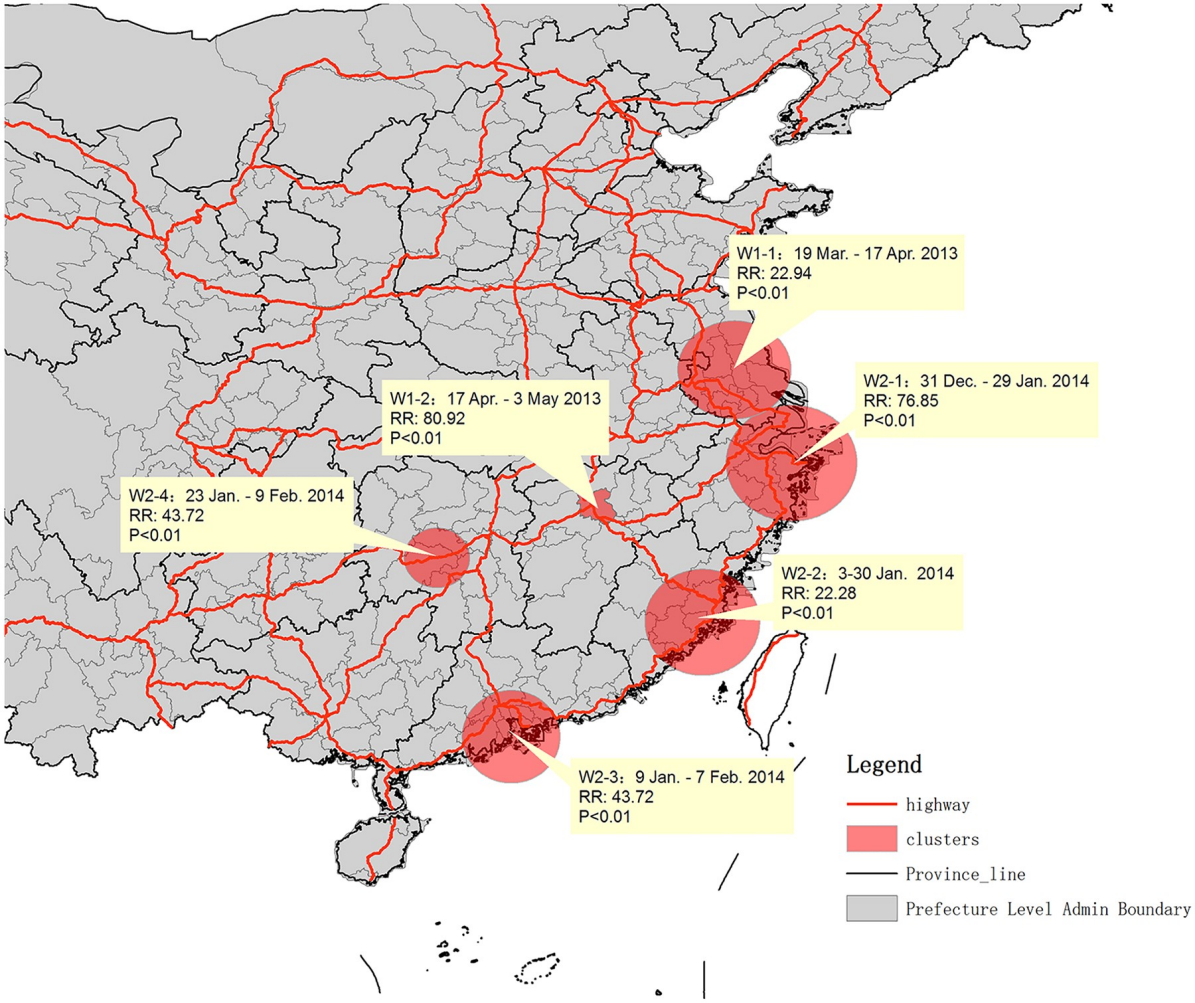
The timing of human case clusters during the first two H7N9 waves. Footnotes: Cluster of W1-1 and W1-2 were in the first wave and W2-1 to W2-4 were in the second wave. The date shows the duration of onset dates of human cases in the clusters.

According to the spatio-temporal analysis of human cases, six clusters were identified. There were two clusters in the first wave (Fig 4: W1-1 and W1-2). One was in Nanjing city and its surrounding area (W1-1) where the onset date of the first patient of this cluster was 19 March 2013, while the other was in Nanchang city (W1-2), Jiangxi province where the onset date of the first patient in the cluster was 17 April 2013.

During the second H7N9 wave, four clusters were identified in north Zhejiang, eastern Fujian, Guangzhou area and central area of Hunan (Fig 4: cluster W2-1 to W2-4). Similarly, onsets of the first cases in the clusters of W2-1, W2-1 and W2-3 were in the early stages (before 10 January 2014) of the second wave, while the cluster W2-4 in Hunan province was formed in the later stage (Fig 4). The onset dates of the first human cases in each cluster were in sequence and the sequence was associated with the distance of following clusters to the original one. It is worth noting that the cluster in Hunan (W2-4) started late January 2014, just 1–2 weeks after increased live poultry movement from the other two clusters in Guangdong and Zhejiang provinces (W2-1 and W2-3) to Hunan province (Figs 3 and 4).

## Discussion

To our knowledge, this is the first study that has presented evidence that closure of live bird markets, adverse publicity and reduced prices has resulted in sudden changes in movements of live poultry during H7N9 influenza epidemics. This has resulted in significant increases in movements to neighboring provinces and other places without human cases at a time when H7N9 influenza virus was at a seasonal peak. In these places biosecurity and movement controls were limited and ineffective in limiting the spread of virus in poultry markets and thus resulted in human cases of the disease. The poultry transportation data from Anhui and Hunan province supports this assumption.

Changes in transport destinations for live yellow chickens may have induced new human clustering in the uninfected areas. Six clusters of human cases were identified, which show that the spatial-temporal distribution of human infection was not random. During the first wave, the first cluster was formed in the early stage and the second one was formed in the late stage. There are no data available to support the opinion that there was poultry transportation from the first clustering area to Nanchang city. However, the first human case in Hunan province in the first wave, whose onset date was 14 April 2013, was exposed to chickens transported from the first clustering area [17, 18]. There is a shortest highway pathway from Nanjing city (center of the cluster W1-1) to Shaoyang city, the location of the first human case in Hunan province, and Nanchang city is between these two cities and on the same highway. The change in poultry movement of the yellow broiler trade platform in Anhui during March to May 2015 may explain the fact that new human cases of H7N9 influenza were reported in Beijing, Henan and Shandong provinces in April 2013 (S1B Fig).

In the second wave, the clusters of human cases moved from eastern China to southern and central China. This is in line with another spatio-temporal study on H7N9 influenza in China [19]. The last human case cluster in Hunan province in the second H7N9 influenza wave started soon after increased poultry importation from the other two clustering areas, which suggests there might be intensive poultry transportation from former cluster areas to new clustering areas. However, transportation data between the clusters in Zhejiang and Fujian (the first two clusters in the second wave) were not available.

H7N9 influenza was spread to many inland provinces and rural areas contributed more cases [16]. Here we also report that more farmers were infected in the second wave than in the first one. This may be due to the extension of H7N9 influenza virus to small cities and rural areas nearby, where LBMs may have lower biosecurity standards and consumers have less awareness about H7N9. There was a media report that panic buying occurred in Maoming city, Guangdong province in April 2013, because of the attractive low price, when many LBMs had closed in neighboring big cities (http://news.ycwb.com/2013-04/14/content_4412992.htm).

Closure of LBMs in areas with human cases had been considered as the most successful measure in H7N9 control. Nonetheless, there are still many obstacles in implementing LBM closure policy. Firstly, citizens living in southern and eastern China prefer fresh chicken meat and this dietary habit is hard to change in a short time [20, 21]. The main reasons for this preference are better flavor with on-site slaughtered meat and concerns about the safety of chilled poultry meat [22]. Secondly, LBM closure policy is not welcomed by most of the stockholders from the poultry industry and many local consumers [23]. Poultry farmers and traders have higher risk of contracting avian-sourced influenza viruses but they perceive H7N9 as a low personal risk [24, 25]. Thirdly, illegal trade is often seen after LBM closure in an area, which makes it even harder for authorities to supervise H7N9 in poultry. Last but not least, LBM closure and disinfection in closed LBMs are found not to be sufficient in eliminating environmental contamination [26]. One health approaches are vital for further improvement of H7N9 control policy. Multidisciplinary studies with joint efforts from the human health sector, veterinary sector, poultry industry and other stakeholders are called for the improvement of H7N9 control in China.

The most challenging part in studying a public health policy from multiple angles is combining pieces of data from different aspects. In this study, we only analyzed the data from the first two waves, because the resolution in open-access data of human and poultry infection had decreased after the first two waves. Nonetheless, this study, providing evidence from a variety of perspectives, provides an important understanding of the risks of spreading H7N9 influenza as a result of long distance transport resulting from LBM closure in China.

## Conclusions

Closure of live bird markets, adverse publicity and reduced prices has resulted in sudden changes in movements of live poultry in waves of H7N9, and these appear to have resulted in new clustering of human cases in neighboring provinces and other uninfected places. Our findings suggest that authorities in uninfected districts should be alert to the risks of sudden changes in movement patterns for live birds after LBM closure or publicity about LBM closure. They should take care to strengthen biosecurity systems and movement controls and to prevent the risks to poultry and humans in their places.

## Supporting information

S1 Table Official actions on LBMs closure in provinces. (DOCX)

S2 Table Trade volume of the platform in Anhui to provinces during February to May 2013. (DOCX)

S1 Fig Yellow broiler price changes in neighboring provinces. Arrows indicate the start dates of LBM closure in provinces with higher prices (Price Unit: RMB/500g).
(TIF)

S2 Fig Human case distributions in different stages in the first two waves. (TIF)

S1 Dataset Original data used in this study. (ZIP)
