# Supplementary material for: Closure of live bird markets leads to the spread of H7N9 influenza in China
Source: PLoS One. 2018 Dec 12;13(12):e0208884. doi: 10.1371/journal.pone.0208884 (PMC6291110; doi:10.1371/journal.pone.0208884)
Supplement: S1 Table — (DOCX) [file pone.0208884.s001.docx]

Appendix 1 Official actions on LBMs closure in provinces

| **Date** | **Province** | **Scope** | **Action** | **Impact score** |
| --- | --- | --- | --- | --- |
| 2013/12/1 | Jiangsu | Capital city | Nanjing government released official document, forbid poultry importation from other provinces. Rest day in LBMs | 5 |
| 2013/12/13 | Jiangsu | City | Yangzhou city reform LBMs in the following 3 years | 4 |
| 2014/1/14 | Zhejiang | City | All the retail spots in supermarkets were closed in Jinhua city and Yiwu city | 5 |
| 2014/1/16 | Zhejiang | City | Xiaoshan district of Hangzhou city forbade imported poultry from outside | 2 |
| 2014/1/20 | Zhejiang | City | Xiaoshan district of Hangzhou city closed LBMs since this date | 5 |
| 2014/1/23 | Jiangsu | City | Suzhou city start to implement the rest day policy in the wholesale LBM named “Nanhuan Bridge”, every Tuesday will be the rest day | 3 |
| 2014/1/24 | Zhejiang | City | All the LBMs in Hangzhou city closed | 8 |
| 2014/1/24 | Zhejiang | City | Shaoxing city suspend poultry trade for 3 weeks， then the LBMs in the urban area will continue to keep closed for 3 months | 5 |
| 2014/1/26 | Zhejiang | City | All the LBMs in urban area of Ningbo city closed since this date | 5 |
| 2014/1/27 | Zhejiang | Whole province | Meeting held by the provincial government, all the cities and counties with more cases should close LBMs for 3 months | 5 |
| 2014/1/29 | Nation wide | National wide | NHFPC suggested to close local LBMs when necessary | 4 |
| 2014/1/30 | Jiangsu | Capital city | Nanjing city all the LBMs closed | 8 |
| 2014/1/31 | Jiangsu | Whole province | Provincial government released an official document to ask local government strengthen H7N9 work and close LBMs when necessary | 3 |
| 2014/2/8 | Zhejiang | City | All the LBMs in urban area of Wenzhou city closed since this date | 5 |
| 2014/2/11 | Zhejiang | City | Jiande city decided to forbid live poultry trade in the key urban areas of the city | 5 |
| 2014/2/11 | Jiangsu | Whole province | One supervision campaign ended, lot of LBMs were affected | 5 |
| 2014/2/13 | Zhejiang | City | Hangzhou city announced to forbid live poultry trade in the key urban area for ever | 5 |
| 2014/1/28 | Guangxi | Whole province | Guangxi provincial government released an official document， to urge local governments to better address the threat of H7N9 | 3 |
| 2014/2/2 | Guangxi | City | The biggest wholesale LBM in Liuzhou city was closed for 5 days | 5 |
| 2014/2/8 | Guangxi | City | 78 LBMs in Liuzhou city would start to imply “rest day” policy henceforth | 3 |
| 2014/1/20 | Fujian | Whole province | Fujian provincial government released an official document ：stop importing poultry from other province and improve LBM management； the poultry trade in one place should be suspended when human infection was confirmed | 8 |
| 2014/1/23 | Fujian | Big city | Xiamen government released an official notification： stop importing poultry from other provinces and other prefectures in Fujian | 5 |
| 2014/1/28 | Fujian | Capital city | Fuzhou government meeting： stop importing poultry from other provinces and other prefectures in Fujian | 5 |
| 2014/1/29 | Fujian | Big city | Haicang district， Xiamen city suspended poultry trade since then， | 5 |
| 2014/2/17 | Anhui | City | Lujiang county closed all the LBMs for 2 weeks | 5 |
| 2014/2/15 | Anhui | City | Huaining and Tongcheng suspended poultry trade | 5 |
| 2014/2/9 | Anhui | City | Susong county, Anqing city closed some LBMs after one human case confirmed | 4 |
| 2014/1/27 | Hunan | Whole province | Forbid poultry importation from positive provinces | 8 |
| 2014/1/29 | Hunan | Capital city | Strengthen LBMs management in Changsha city | 5 |
| 2014/1/31 | Hunan | City | Closure of LBMs in Yongzhou city | 5 |
| 2014/2/6 | Hunan | City | Closure of LBMs in Miluo city | 5 |
| 2014/2/8 | Hunan | Whole province | Provincial government meeting on LBM management | 5 |
| 2013/12/23 | Guangdong | Whole province | The provincial government asked 3 times rest day in all the LBMs in GD before the spring festival | 5 |
| 2014/1/3 | Guangdong | Capital city and 1 other city | Temporal LBMs closure in Guangzhou and Zhuhai city | 8 |
| 2014/1/13 | Guangdong | City | Foshan started to close LBMs in several districts of the city | 5 |
| 2014/1/23 | Guangdong | Capital city | All the LBMs closed for 1 day | 5 |
| 2014/1/27 | Guangdong | City | All the wholesale LBM in Shenzhen closed for 1 day | 4 |
| 2014/1/31 | Guangdong | City | All the LBMs in Shenzhen closed for 2 weeks | 6 |
| 2014/1/31 | Shanghai | Whole province | All the LBMS closed for 3 weeks | 10 |
