# Supplementary material for: Closure of live bird markets leads to the spread of H7N9 influenza in China
Source: PLoS One. 2018 Dec 12;13(12):e0208884. doi: 10.1371/journal.pone.0208884 (PMC6291110; doi:10.1371/journal.pone.0208884)
Supplement: S2 Table — (DOCX) [file pone.0208884.s002.docx]

**S2 Table. Trade volume of the platform in Anhui to provinces during**

**February to May 2013**

| **Destination** | **February** | **March** | **April** | **May** | **Total** |
| --- | --- | --- | --- | --- | --- |
| **Anhui** | 0 | 3700 | 51 | 0 | 3751 |
| **Fujian** | 0 | 0 | 4000 | 0 | 4000 |
| **Guangdong** | 35350 | 52900 | 107885 | 0 | 196135 |
| **Hebei** | 0 | 3900 | 88700 | 0 | 92600 |
| **Henan** | 40120 | 42550 | 104140 | 16450 | 203260 |
| **Hubei** | 28400 | 42600 | 4400 | 0 | 75400 |
| **Hunan** | 13200 | 44580 | 25060 | 0 | 82840 |
| **Jiangsu** | 5736 | 83250 | 32660 | 19400 | 141046 |
| **Jiangxi** | 6600 | 27800 | 5900 | 0 | 40300 |
| **Shandong** | 7000 | 18600 | 690100 | 117450 | 833150 |
| **Shanghai** | 7920 | 11590 | 1100 | 0 | 20610 |
| **Zhejiang** | 4000 | 4300 | 2000 | 0 | 10300 |
| **Chongqing** | 0 | 2400 | 0 | 0 | 2400 |
