# Supplementary figures and images for: Closure of live bird markets leads to the spread of H7N9 influenza in China
Source: PLoS One. 2018 Dec 12;13(12):e0208884. doi: 10.1371/journal.pone.0208884 (PMC6291110; doi:10.1371/journal.pone.0208884)

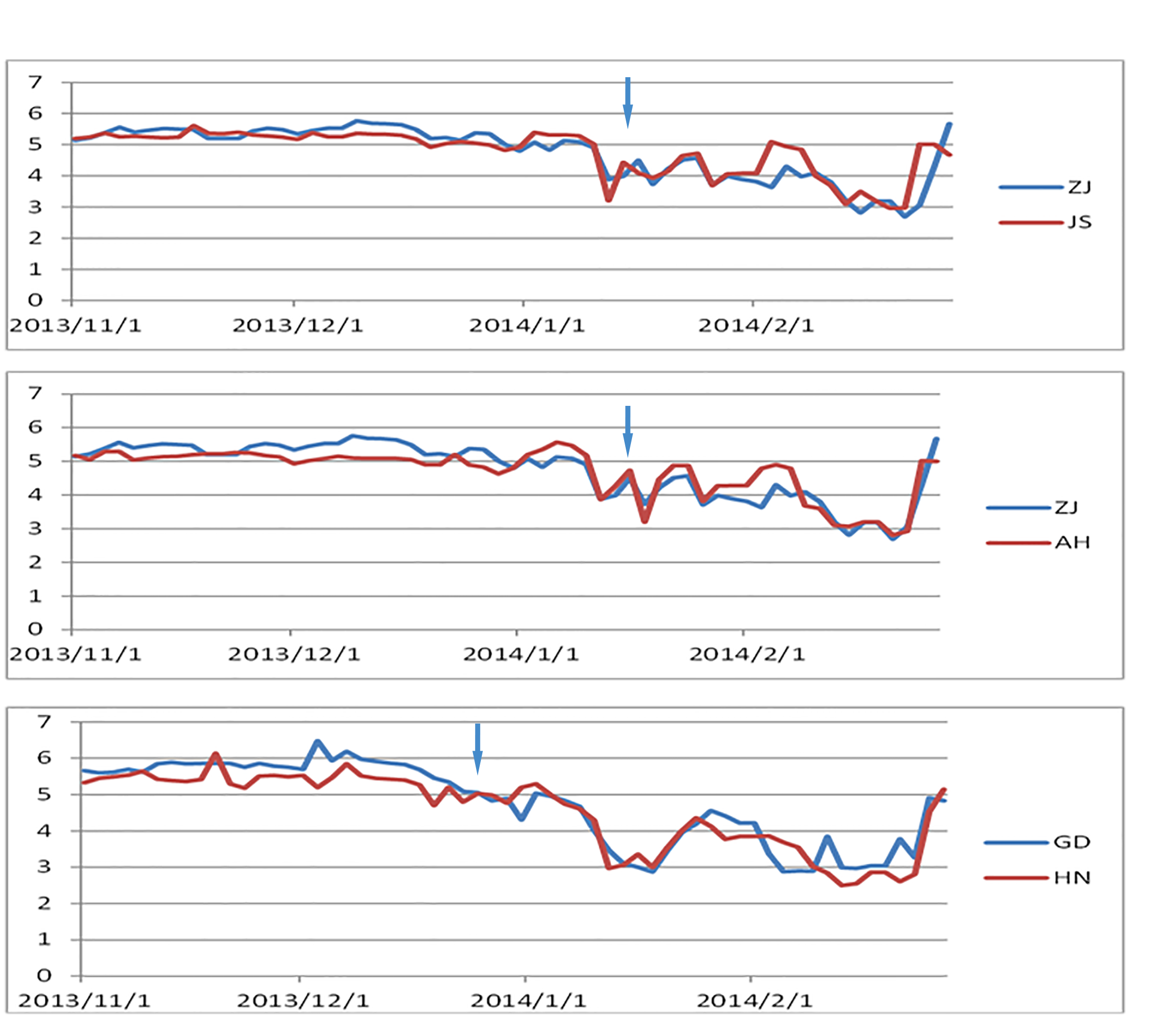

Supplement: S1 Fig — Arrows indicate the start dates of LBM closure in provinces with higher prices (Price Unit: RMB/500g). (TIF) [file pone.0208884.s003.tif]

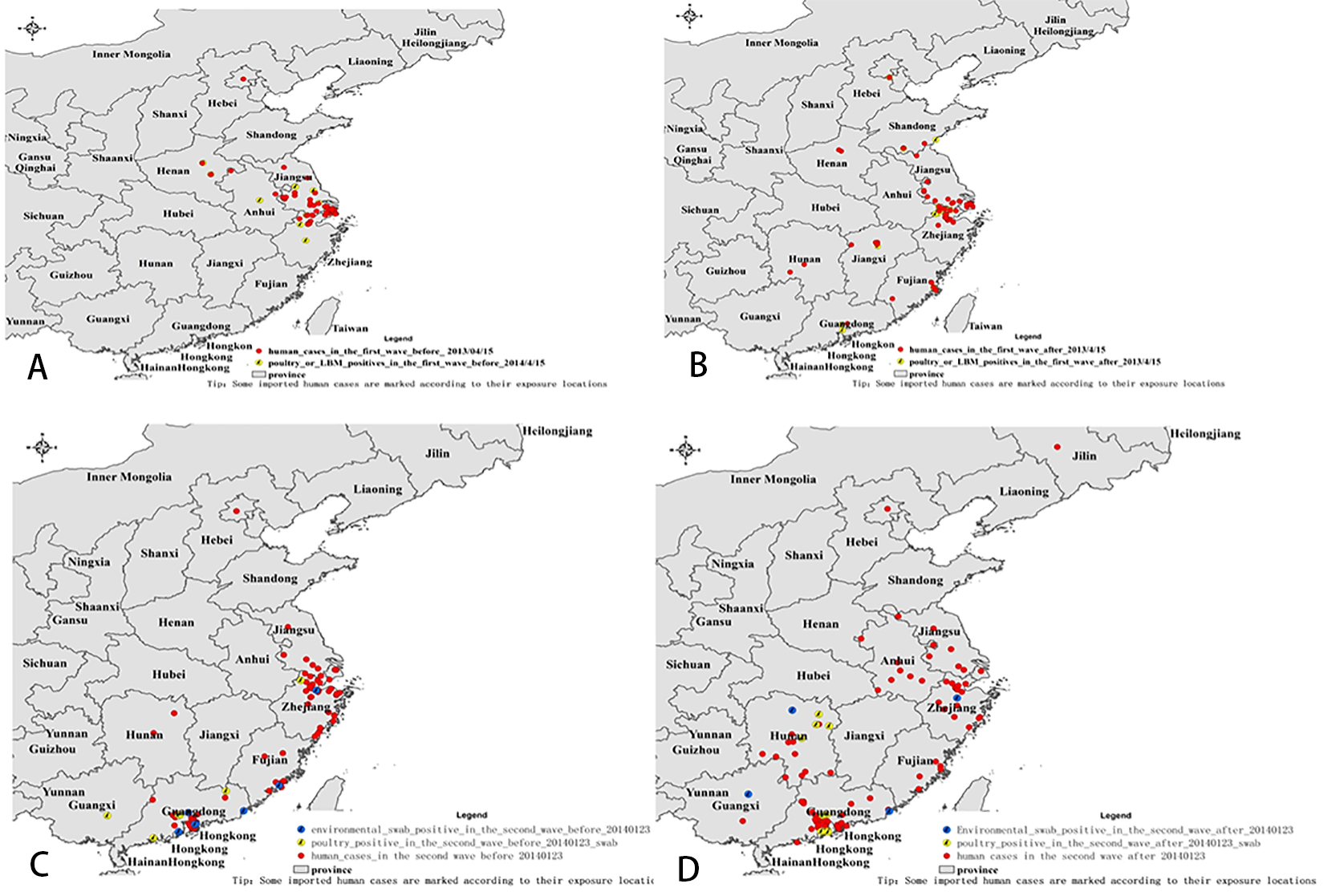

Supplement: S2 Fig — (TIF) [file pone.0208884.s004.tif]
